# *Ruta chalepensis* L. In Vitro Cultures as a Source of Bioactive Furanocoumarins and Furoquinoline Alkaloids

**DOI:** 10.3390/life13020457

**Published:** 2023-02-06

**Authors:** Agnieszka Szewczyk, Mariusz Grabowski, Dominika Zych

**Affiliations:** 1 Department of Pharmaceutical Botany, Faculty of Pharmacy, Jagiellonian University Medical College, 30-688 Krakow, Poland; 2 SSG of Medicinal Plants and Mushroom Biotechnology Department of Pharmaceutical Botany, Jagiellonian University Medical College, 30-688 Krakow, Poland

**Keywords:** *Ruta chalepensis*, bioreactor, in vitro cultures, linear furanocoumarins, furoquinoline alkaloids, plant biotechnology

## Abstract

**Simple Summary:**

Of late, the interest in alternative sources of bioactive compounds to field crops has been increasing due to decreasing crop acreages and climate change. Plant biotechnology offers the possibility of cultivating valuable medicinal plant species using in vitro methods. In vitro cultures are characterized by a significant increase in biomass, and one of their primary advantages is that they are independent of climate and soil conditions. This study aimed to investigate the ability of *Ruta chalepensis* bioreactor cultures to produce the following valuable bioactive substances: linear furanocoumarins and furoquinoline alkaloids. Bioreactor cultures of *R. chalepensis* may be used as a good raw material for the production of important bioactive compounds from the groups of coumarins and alkaloids.

**Abstract:**

Recently, due to the decreasing areas of cultivation and climate change, the use of biotechnological methods to obtain biomass, which is a source of valuable bioactive metabolites, is becoming more and more interesting. In this study, *Ruta chalepensis* in vitro cultures were investigated in RITA^®^ temporary immersion bioreactors. Biomass growth and the production of secondary metabolites in 4- and 5-week growth cycles on three variants of the Linsmaier and Skoog (LS) medium (naphthyl-1-acetic acid/6-benzylaminopurine (NAA/BAP): 0.5/1.0, 0.1/0.1, and 1.0/1.0 mg/L) were analyzed. Using high-performance liquid chromatography of methanolic extracts of biomass, the presence of linear furanocoumarins (bergapten, isoimperatorin, isopimpinellin, psoralen, and xanthotoxin) and furoquinoline alkaloids (γ-fagarine, 7-isopentenyloxy-γ-fagarine, and skimmianine) was confirmed. The highest content of linear furanocoumarins (1170 mg/100 g DW (dry weight)) was observed in the LS medium variant containing 0.5/1.0 mg/L NAA/BAP (4-week growth cycle). The highest content of furoquinoline alkaloids (449 mg/100 g DW) was observed in the LS medium variant containing 0.1/0.1 mg/L NAA/BAP (5-week growth cycle). Hence, *R. chalepensis* bioreactor cultures may be used as a biotechnological source of linear furanocoumarins (xanthotoxin and bergapten) and furoquinoline alkaloids (skimmianine and γ-fagarine).

## 1. Introduction

Besides *Ruta graveolens*, the most valuable species of the *Ruta* genus is *Ruta chalepensis*. *R. chalepensis* L. (Rutaceae) is native to the Mediterranean region but widely distributed and cultivated worldwide in temperate and tropical climates, e.g., Latin America, Africa (Tunisia, Algeria, Libya), and Asia (Israel, Iran, Turkey, Syria) [1,2,3]. *R. chalepensis* contains secondary metabolites from various chemical groups as follows: alkaloids (acridine, quinoline, furoquinoline alkaloids), flavonoids, coumarins (furanocoumarins, dihydrofuranocoumarins, coumarin dimers, simple coumarins), and essential oils (primary compounds: nonan-2-one and undecan-2-one) [4,5,6,7]. *Ruta* is one of the most frequently used plants for medicinal purposes [1]. *R. chalepensis* exhibits a wide range of healing effects: antioxidant, anti-inflammatory, antipyretic, spasmolytic, sedative, analgesic, antibacterial, antifungal, cytotoxic, and antidiabetic effects, to name a few [8,9,10,11,12,13,14]. The herb of *R. chalepensis* has linear furanocoumarins (xanthotoxin and bergapten) that are used in dermatology. Furanocoumarins are responsible for photoreactive properties, so they are used as pigmentation stimulators and antiproliferative factors. The so-called PUVA therapy (psoralens and UVA irradiation) is a kind of photochemotherapy used in the treatment of vitiligo, psoriasis, mycosis, and atopic eczema [15,16,17,18]. The use of furanocoumarins needs to be closely monitored as they can also lead to side effects (photodermatoses and kidney and liver damage) [18]. There are also reports of other applications of furanocoumarins. Bergapten shows activity as a selective blocker of axolemmal potassium channels and may be useful in the treatment of demyelinating diseases [1,19]. Furanocoumarins exhibit anticancer activity and are currently considered novel mechanism-based drug candidates for the treatment of selected forms of cancer [20]. Commercial production of furanocoumarins via chemical synthesis is quite expensive, and they are obtained from bergamot oil (*Citrus bergamia*). Unfortunately, the areas of cultivation of this plant are gradually decreasing. Hence, there is a need for new sources of these compounds. The genus *Ruta* is a promising source, but scaling up field crops is similarly problematic [19]. Species of the genus *Ruta* are also rich in furoquinoline alkaloids (γ-fagarine and skimmianine). Furoquinoline alkaloids have antifungal and antibacterial activities, and acetylcholinesterase-inhibiting properties, as well as an inhibitory effect on the 5-HT2 receptor [21,22].

In vitro cultures can be used as a source of secondary metabolites. In vitro plant cultures have a significant potential for the commercial production of secondary metabolites. They allow for the synthesis of bioactive metabolites under controlled conditions, thus making producers independent of climate and soil conditions. They can be carried out continuously, and the biomass obtained is homogenous. Cell lines with favorable characteristics, e.g., high production of selected metabolites, can be selected, which are a valuable and reliable source of secondary metabolites [23,24]. The production of secondary metabolites in in vitro cultures is also associated with a few problems. The major problem is scaling up for industrial production. On the one hand, shoot cultures are the most productive as they retain a high degree of tissue differentiation; on the other hand, carrying out this type of culture on a larger scale becomes difficult. Temporary immersion system bioreactors are extremely useful for this purpose. They allow for the rapid multiplication of biomass and are easy to use. Our previous studies on in vitro agitated shoot cultures of *R. chalepensis* confirmed the high biosynthetic potential of these cultures in terms of linear furanocoumarins and furoquinoline alkaloids [25].

With the encouraging findings of these studies, this study attempted to scale up the cultures carried out using the temporary immersion system and assess the usefulness of this method for the commercial production of the abovementioned metabolites. This type of culture is conducted for the first time for the species *R. chalepensis*.

## 2. Materials and Methods

### 2.1. In Vitro Cultures

The starting material used in this study was the in vitro cultures of *R. chalepensis*, established in 2018 from the seeds obtained from the Botanical Garden of Maria Skłodowska-Curie University, Lublin, Poland. The initial cultures were carried out as liquid stationary cultures on the Linsmaier and Skoog (LS) medium [26] and the following plant growth and development regulators: naphthyl-1-acetic acid (NAA) and 6-benzylaminopurine (BAP) (1.0/1.0 mg/L).

*R. chalepensis* shoot cultures were carried out in RITA^®^ bioreactors (VITROPIC, Saint-Mathieu-de-Treviers, France). Of the previously grown plant biomass, 5.0 g was placed in the bioreactors, to which 200 mL of the LS medium was added. Growth and development regulators in three concentration variants (NAA/BAP: 0.5/1.0, 0.1/0.1, and 1.0/1.0 mg/L) were then added to it. Immersion frequency was 5 min every 90 min. Cultivation was carried out for a period of 4 and 5 weeks. Finally, fresh biomass was collected and dried at approximately 38 °C.

### 2.2. Extraction

Micronized dry biomass (1.0 g) was weighed into 250 mL round-bottom flasks. Biomass was then extracted with methanol (50 mL) at the solvent boiling point (64.7 °C). Following this, the extracts were evaporated, dissolved in 4.0 mL high-performance liquid chromatography (HPLC)-grade methanol, and filtered through Millipore filters (pore size 0.22 µm) for the HPLC analysis.

### 2.3. HPLC Analyses

HPLC analysis was carried out as previously described [27] on a liquid chromatograph (LaChrom Elite, Hitachi, Tokyo, Japan) using DAD detection (200–400 nm). Quantification was carried out at two wavelengths (λ): 254 and 330 nm.

Reference substances were obtained from bergapten, imperatorin, xanthotoxin, and psoralen from Roth (Karlsruhe, Germany); 5,7-dimethoxycoumarin, 4-hydroxy-6-methylcoumarin, 6-methylcoumarin, osthole, and umbelliferone from Sigma-Aldrich (St Louis, MO, USA); coumarin and scopoletin from Fluka (Bucha, Switzerland); 4-methylumbelliferone, 4,6-dimethoxy-2H-1-benzopyran-2-one, and skimmianine from ChromaDex (Irvine, CA, USA); and isopimpinellin, isoimperatorin, daphnetin 7-methyl ether, rutaretin, daphnetin, osthenol, bergaptol, daphnetin dimethyl ether, γ-fagarine, and 7-isopentenyloxy-γ-fagarine from ChemFaces (Wuhan, China).

### 2.4. Statistical Analysis

Statistical analysis of the results was carried out using analysis of variance. The NIR post-hoc test was used, and the comparison of homogeneous groups was made, with two independent variables (growth cycle and medium variant). STATISTICA software (version 13.3, StatSoft Inc., Tulsa, OK, USA) was used. Detailed results of the statistical analysis are included in the Supplementary Materials.

## 3. Results

### 3.1. In Vitro Cultures

**Figure 1:**
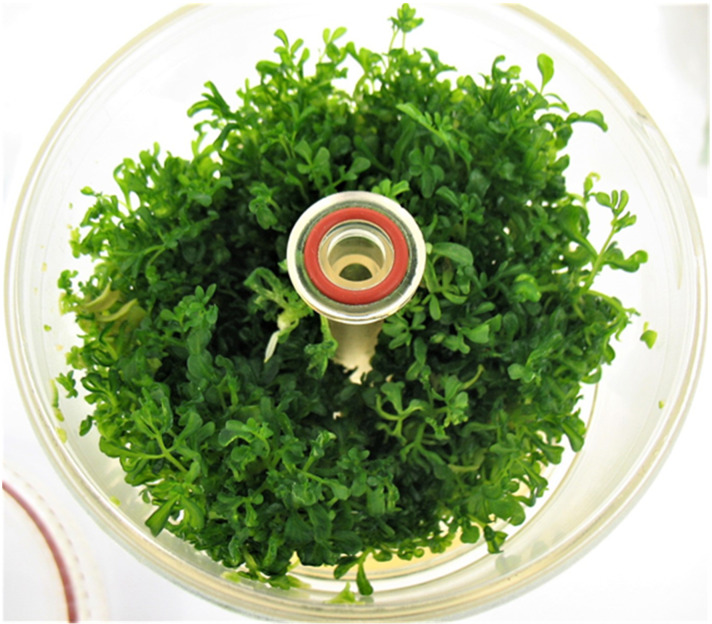
*Ruta chalepensis* bioreactor culture (LS NAA/BAP 0.1/0.1 mg/L, 4-week growth cycle).

The in vitro cultures of *R. chalepensis* were grown as shoot cultures in 4- and 5-week growth cycles on three variants of the LS medium (LS NAA/BAP 0.5/1.0, 0.1/0.1, and 1.0/1.0) in RITA^®^ bioreactors. They were characterized by intense green color and grew primarily in the form of shoots with a slight tendency to form callus tissue (Figure 1). The increase in biomass in all cultures was similar. Their dry biomass content ranged between 3.97 (the LS medium containing 0.1/0.1 mg/L NAA/BAP, 4-week growth cycle) and 3.17 g (the LS medium containing 1.0/1.0 mg/L NAA/BAP, 4-week growth cycle). The highest weight was obtained after the 4-week cultivation period (growth cycle) on the LS medium containing 0.1/0.1 mg/L NAA/BAP. No significant differences in biomass growth were observed between 4- and 5-week growth cycles. The obtained dry biomass content is summarized in Table 1.

### 3.2. HPLC Analyses

#### 3.2.1. Production of Linear Furanocoumarins

HPLC analyses of methanol extracts from the biomass of *R. chalepensis* bioreactor cultures confirmed the presence of five linear furanocoumarins: bergapten, isoimperatorin, isopimpinellin, psoralen, and xanthotoxin. A sample HPLC chromatogram of the culture extract is included in Figure S1 of the Supplementary Materials.

The total content of five linear furanocoumarins determined in the culture extracts during the 4-week growth cycle was quite higher in comparison with the 5-week growth cycle and ranged between 756 and 1170 mg/100 g DW (dry weight). This higher accumulation of furanocoumarins was attributable to the use of a medium containing lower concentrations of plant growth and development regulators (NAA/BAP 0.5/1.0 and 0.1/0.1 mg/L). The primary compound was xanthotoxin, with the highest content of 604 mg/100 g DW on the LS medium variant containing 0.1/0.1 mg/L NAA/BAP. Other major furanocoumarins are bergapten (highest content 245 mg/100 g DW, LS NAA/BAP 0.5/1.0) and psoralen (highest content 222 mg/100 g DW, LS NAA/BAP 0.5/1.0). Isopimpinellin and isoimperatorin were accumulated in lower amounts (76 and 54 mg/100 g DW, respectively).

In the 5-week growth cycle, the total content of linear furanocoumarins in the biomass extracts ranged between 841 and 921 mg/100 g DW. The content of individual furanocoumarins was generally lower in comparison with the 4-week growth cycle (Table 2).

**Figure 2:**
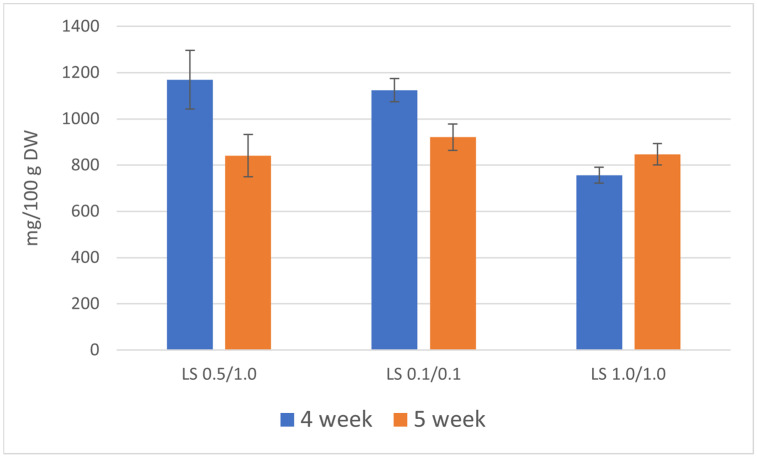
Total content of furanocoumarins [mg/100 g DW] in the biomass of *R. chalepensis* maintained in RITA^®^ bioreactors, depending on the duration of the growth cycle of the culture (4 and 5 weeks) and LS medium variant NAA/BAP (LS 0.5/1.0, 0.1/0.1, and 1.0/1.0).

The LS medium containing 0.5/1.0 mg/L NAA/BAP seemed to be the most advantageous for the production of bioactive furanocoumarins. However, there were no significant differences between LS 0.5/1.0 and 0.1/0.1 variants. In principle, both variants can be used to produce bioactive furanocoumarins. The LS medium with the highest concentration of plant growth and development regulators (LS 1.0/1.0) was the least favorable and cannot be considered for the cultivation of *R. chalepensis* bioreactor cultures. To increase the content of linear furanocoumarins, 4-week growth cycles are preferable (Figure 2).

#### 3.2.2. Production of Furoquinoline Alkaloids

HPLC analyses of methanol extracts from the biomass of *R. chalepensis* bioreactor cultures confirmed the presence of three furoquinoline alkaloids: γ-fagarine, 7-isopentenyloxy-γ-fagarine, and skimmianine. A sample HPLC chromatogram is included in Figure S1 of the Supplementary Materials.

The total content of the three furoquinoline alkaloids determined in the culture extracts during the 4-week growth cycle was lower in comparison with the 5-week growth cycle and ranged between 214 and 326 mg/100 g DW. This higher accumulation of furoquinoline alkaloids was attributable to the use of a medium containing the lowest concentrations of growth and development regulators (NAA/BAP 0.1/0.1 mg/L). During the 5-week growth cycle, the total content of these three furoquinoline alkaloids ranged between 305 and 449 mg/100 g DW.

The primary compound was skimmianine, with the highest content of 292 mg/100 g DW on the LS medium variant containing 0.1/0.1 mg/L NAA/BAP (5-week growth cycle), followed by γ-fagarine (highest content 187 mg/100 g DW, LS NAA/BAP 0.1/0.1, 4-week growth cycle). 7-isopentenyloxy-γ-fagarine was accumulated in lower amounts (highest content 17 mg/100 g DW, LS NAA/BAP 0.1/0.1, 4-week growth cycle) (Table 3).

**Figure 3:**
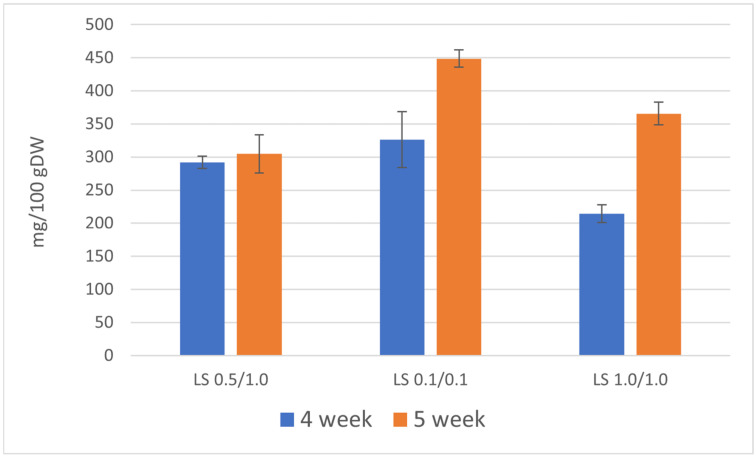
Total content of furoquinoline alkaloids [mg/100 g DW] in the biomass of *R. chalepensis* maintained in RITA^®^ bioreactors, depending on the duration of the growth cycle of culture (4 and 5 weeks) and LS medium variant NAA/BAP (LS 0.5/1.0, 0.1/0.1, 1.0/1.0).

Higher content of furoquinoline alkaloids can be achieved by using the medium containing 0.1/0.1 mg/L NAA/BAP. LS media containing a higher concentration of plant growth and development regulators (LS 0.5/1.0 and 1.0/1.0) were less favorable for the production of furoquinoline alkaloids. To achieve a higher total content of furoquinoline alkaloids, 5-week growth cycles are preferable (Figure 3). However, analysis of the results for individual compounds showed that only skimmianine is produced in higher amounts during the 5-week growth cycle and the 4-week growth cycle is more favorable for γ-fagarine production (Table 3).

## 4. Discussion

So far, *R. chalepensis* in vitro cultures have not been studied in detail. Studies on in vitro callus agar cultures of *R. chalepensis* showed the presence of furanocoumarins such as bergapten and isopimpinellin. Furthermore, the presence of alkaloids such as skimmianine, γ-fagarine, rutacridone, rutacridone epoxide, gravacridone, and arborinin was reported. However, researchers have not quantified these metabolites [28].

*R. chalepensis* suspension cultures produced three metabolites: isorutarin, rutarensin, and 3-hydroxy-3-methylglutaric acid. Similarly, these metabolites have not been quantified [29].

Studies on the effect of LED light on the accumulation of the four coumarins—xanthotoxin, bergapten, psoralen, and umbelliferone—have shown that blue light increases the accumulation of these metabolites in *R. chalepensis* callus cultures. However, the content of these compounds was significantly lower than that of the present study. In addition, quantitative determinations were carried out on the fresh weight (FW) in the abovementioned studies, whereas in the present study, the chemical composition of the DW was analyzed. The highest content of xanthotoxin was 0.95 mg/100 g FW, and the bergapten content was higher than the xanthotoxin content and amounted to 48.9 mg/100 g FW [20].

The abovementioned studies also investigated other types of cultures (callus and suspension cultures). These cultures have a low degree of tissue differentiation, which are characterized by lower levels of secondary metabolite accumulation. In the present study, another type of in vitro culture was used—shoot culture. Due to the higher level of tissue differentiation, a higher biosynthetic potential can be observed in these cultures. Our previous studies on agitated shoot cultures of *R. chalepensis* (the LS medium variant NAA/BAP 0.1/0.1 mg/L) showed that the metabolism of shoot cultures is directed toward the production of furanocoumarins and furoquinoline alkaloids [25]. The results of the present study also showed a similar ability of cultures grown in bioreactors. In our previous studies, the content of the primary furanocoumarins—xanthotoxin and bergapten—was extremely high (509 and 281 mg/100 g DW, respectively), which is comparable to the results obtained in the present study (highest content 604 and 245 mg/100 g DW, respectively). However, the content of the primary alkaloids followed a different trend. The dominant compound was γ-fagarine, and skimmianine was accumulated in a lower amount, which is contrary to the present study. The content of these alkaloids was lower (γ-fagarine 79 mg/100 g DW and skimmianine 56 mg/100 g DW) than in the present study (187 and 292 mg/100 g DW, respectively) [25].

In vitro cultures may differ from their parent plants in the composition of secondary metabolites. Studies on in vitro cultures of three species of rue—*R. graveolens*, *R. chalepensis,* and *R. corsica* showed that the dominant secondary metabolites were compounds from the group of furanocoumarins and alkaloids, while phenolic compounds were accumulated in small amounts [25]. This observation also applies to the cultures in the present study. The content of some metabolites (furanocoumarin) was extremely high, higher than in the parent plant. On the other hand, other compounds (e.g., phenolics) were accumulated in much lower amounts. In addition, HPLC analyses did not show the presence of flavonoids, including rutoside—a characteristic flavonoid found in the genus *Ruta*. This may result from damage to the metabolic pathway or inhibition of the expression of certain genes [23].

In this study, *R. chalepensis* shoot cultures in the temporary immersion bioreactor RITA^®^ were carried out for the first time. RITA^®^ and other temporary immersion bioreactors (Plantform^™^) have recently been used to produce secondary metabolites. They are highly useful for cultivating shoot cultures. This method allows the large-scale production of secondary metabolites from various groups of compounds. Studies on *Schizandra chinensis* cultures carried out in various types of bioreactors showed the high production of lignans in cultures carried out in Plantform^™^ and RITA^®^ bioreactors [30]. *Centella asiatica* cultures in RITA^®^ and Plantform^™^ bioreactors were a valuable source of asiaticoside, phenolic acids, and flavonoids [31]. *Leucojum aestivum* cultures maintained in RITA^®^ bioreactors produced a high content of Amaryllidaceae alkaloids, especially galanthamine and lycorine [32].

The *Ruta* genus has long been of interest due to its diverse chemical composition and multidirectional healing effect. Therefore, obtaining its bioactive metabolites is of interest [33]. In vitro cultures of the most well-known and studied species *Ruta graveolens* have been intensively studied for many years. Studies of the chemical composition of *R. graveolens* cultures resulted in the isolation of many metabolites from the following groups: coumarins (22 compounds), quinoline alkaloids (13 compounds), acridone alkaloids (17 compounds), and almost 50 essential oil components [34]. Many publications have focused on the possibilities of increasing the production of secondary metabolites in in vitro cultures of *R. graveolens* using various strategies known in plant biotechnology, such as changing the light conditions, elicitation, and feeding with phenylalanine. The most dominant furanocoumarins were xanthotoxin and bergapten, and the most dominant furoquinoline alkaloids were skimmianine and γ-fagarine [35,36,37,38]. Studies on stationary liquid cultures of *R. graveolens* conducted in various light conditions on LS medium containing 2/2 mg/L NAA/BAP (6-week growth cycle) showed the presence of five furanocoumarins: psoralen, xanthotoxin, isopimpinellin, bergapten, and imperatorin. Additionally, the content of umbelliferon was analyzed. The highest total content of coumarins (1022 mg/100 g DM) was detected in the cultures grown under white constant artificial light. Two main furanocoumarins were xanthotoxin and bergapten with the highest content of 433 and 219.5 mg/100 g DM, respectively [35]. In the subsequent studies on *R. graveolens* cultures, it was observed that the use of biotic and abiotic elicitors is a promising strategy to increase the production of furanocoumarins. The effect of abiotic elicitors (benzothiazole and saccharin) was studied in agitated shoot cultures of *R. graveolens* using B_5_ medium (4-week growth cycle). It was observed that the addition of benzothiazole in a concentration of 5% increased the production of furanocoumarins: xanthotoxin (288.36 mg/100 g DW, 8.5-fold increase, compared to control cultures), bergapten (153.78 mg/100 g DW, 3.7-fold increase), and isopimpinellin (78.9 mg/100 g DW, 14-fold increase); additionally, the cultures accumulated psoralen (82 mg/100 g DW), which was not found in the control samples [36]. Elicitation with another elicitor, chitin, also showed a positive effect on the production of furanocoumarins, but the obtained contents were slightly lower: xanthotoxin (212 mg/100 g DW, 6.3-fold increase, compared to control cultures), bergapten (146 mg/100 g DW, 3.5-fold increase), isopimpinellin (61 mg/100 g DW, 10.9-fold increase), and psoralen (68 mg/100 g DW, not detected in control cultures) [37]. The worst results were obtained using a bacterial (*Bacillus* sp.) lysate as an elicitor: xanthotoxin (153 mg/100 g DW, 5-fold increase, compared to control cultures), bergapten (90 mg/100 g DW, 2-fold increase), isopimpinellin (49 mg/100 g DW, 9-fold increase), and psoralen (52 mg/100 g DW, not detected in control cultures) [38].

Elicitation also had the effect of increasing the production of furoquinoline alkaloids, but the type of elicitor used had the opposite effect on alkaloid production compared to the accumulation of furanocoumarins. The addition of benzothiazole increased the production of three alkaloids: γ-fagarine (5.8 mg/100 g DW, 12-fold increase, compared to control cultures), kokusaginine (2.8 mg/100 g DW, 5.3-fold increase), and skimmianine (6.4 mg/100 g DW, 15.7-fold increase) [36]. Chitin, in this case, was more effective and increased production of γ-fagarine (12.6 mg/100 g DW, 36-fold increase), kokusaginine (4.4 mg/100 g DW, 9-fold increase), and skimmianine (14.7 mg/100 g DW, 25-fold increase). In addition, the cultures produced dictamnine (1.04 mg/100 g DW), which was not found in the control samples [37]. The best results were obtained using a bacterial lysate from *Pectobacterium atrosepticum*. The content of furoquinoline alkaloids was as follows: γ-fagarine (68.0 mg/100 g DW), kokusaginine (17.2 mg/100 g DW), skimmianine (48.0 mg/100 g DW), and dictamnine (9.9 mg/100 g DW) [38].

Subsequent research focused on another strategy for increasing the production of secondary metabolites in in vitro cultures of *R. graveolens* (agitated shoot cultures)—precursor feeding of the metabolic pathway. As a precursor, phenylalanine was used at a concentration of 1.25 g/L. The method used allowed for an increase in the production of phenolic acids and catechins, but no significant effect of phenylalanine on the production of furanocoumarins and furoquinoline alkaloids was observed. The highest concentration of furanocoumarins was as follows: 482.5 mg/100 g DW (xanthotoxin), 392.6 mg/100 g DW (bergapten), and 1059.9 mg/100 g DW (total content of furanocoumarins). Furoquinoline alkaloids were accumulated in the following amounts: 101.4 mg/100 g DW (skimmianine), 94.5 mg/100 g DW (γ-fagarine), and 185.6 mg/100 g DW (total content of furoquinoline alkaloids) [39].

In the study on *R. graveolens*-agitated cultures, the content of most dominant coumarins was as follows: xanthotoxin (428.3 mg/100 g DW) and bergapten (186.6 mg/100 g DW). The maximum total content (917.2 mg/100 g DW) of linear furanocoumarins (xanthotoxin, bergapten, isoimperatorin, isopimpinellin, and psoralen) was reached after a 5-week growth cycle on LS medium containing 0.1/0.1 mg/L NAA/BAP. The maximum total content of furoquinolic alkaloids was 156 mg/100 g DW. The main compounds were skimmianine (94.6 mg/100 g DW) and γ-fagarine (54.5 mg/100 g DW) [25].

The results of the current research have shown that in vitro cultures of *R. chalepensis* are characterized by a similar chemical composition to cultures of *R. graveolens*. However, the highest concentrations of main compounds obtained are higher than those in *R. graveolens* cultures. This may be due to the technique of cultivating cultures in RITA® bioreactors. Based on the obtained results, *R. chalepensis* bioreactor cultures can be a good raw material for the production of linear furanocoumarins. Xanthotoxin and bergapten are used in dermatology, and bergapten shows fewer secondary effects than xanthotoxin [19]. These studies have confirmed an extremely high content of xanthotoxin and bergapten in *R. chalepensis* cultures (above 600 and 245 mg/100 g DW, respectively). For instance, the content of these compounds in the parent plants can reach the highest values of 10 mg/100 g DW (xanthotoxin) and 260 mg/100 g DW (bergapten) [19].

*R. chalepensis* bioreactor cultures are also rich in the furoquinoline alkaloids γ-fagarine and skimmianine (highest content about 200 and 300 mg/100 g DW, respectively), which is interesting from a practical point of view.

For the production of bioactive furanocoumarins, the LS medium variant containing 0.5/1.0 mg/L NAA/BAP seemed to be the most advantageous. A higher content of alkaloids can be achieved using the variant containing 0.1/0.1 mg/L NAA/BAP. The majority of the metabolites are accumulated in higher amounts in the 4-week growth cycle, except for skimmianine, which was accumulated in higher amounts after the 5-week growth cycle.

## 5. Conclusions

The results of this study suggest that *R. chalepensis* bioreactor cultures can be a source of linear furanocoumarins and furoquinoline alkaloids. The bioreactor cultures showed good biomass growth, and the most favorable increase in biomass was observed during the 4-week growth cycle. The optimal medium to obtain biomass was found to be the LS medium containing 0.1/0.1 mg/L NAA/BAP. Variants containing lower concentrations of plant growth and development regulators NAA/BAP (0.5/1.0 and 0.1/0.1 mg/L) promoted a higher accumulation of secondary metabolites. These results encourage further research into the possibilities for the large-scale production of bioactive compounds in *R. chalepensis* cultures, e.g., in Plantform^™^ bioreactors. Strategies to increase the content of bioactive metabolites in in vitro cultures, such as elicitation and feeding with metabolic pathway precursors, can also be used. An interesting step in research could also be to investigate the effect of other plant growth and development regulators on the accumulation of secondary metabolites in bioreactor cultures of *R. chalepensis*.

## Figures and Tables

**Table 1:** Dry weight [g] obtained from *R. chalepensis* cultures maintained in RITA^®^ bioreactors, depending on the duration of the growth cycle of culture (4 and 5 weeks) and LS medium variant NAA/BAP (0.5/1.0, 0.1/0.1, and 1.0/1.0 mg/L). Mean of three replications ± SD.

LS Medium Variant	Dry Weight [g]	
NAA/BAP mg/L	4-Week Growth Cycle	5-Week Growth Cycle
0.5/1.0	3.56 ± 0.26	3.66 ± 0.12
0.1/0.1	3.97 ± 0.46	3.93 ± 0.12
1.0/1.0	3.17 ± 0.14	3.46 ± 0.16

**Table 2:** Contents of furanocoumarins [mg/100 g DW] obtained from *R. chalepensis* cultures maintained in RITA^®^ bioreactors, depending on the duration of the growth cycle of culture (4 and 5 weeks) and LS medium variant NAA/BAP (0.5/1.0, 0.1/0.1, 1.0/1.0 mg/L). Means of three repetitions ± SD. Letters denote homogeneous groups. Different letters indicate significant differences (*p* < 0.05).

Metabolite	LS Medium Variant	Content [mg/100 g DW]	
	NAA/BAP mg/L	4-Week Growth Cycle	5-Week Growth Cycle
Xanthotoxin	0.5/1.0	592.57 ± 68.05 ^a^	425.23 ± 45.48 ^bcd^
	0.1/0.1	603.96 ± 22.33 ^a^	486.72 ± 34.18 ^cd^
	1.0/1.0	375.43 ± 25.46 ^bc^	444.87 ± 28.55 ^bcd^
Bergapten	0.5/1.0	244.76 ± 25.73 ^a^	136.17 ± 12.94 ^c^
	0.1/0.1	196.89 ± 6.08 ^b^	129.81 ± 6.67 ^c^
	1.0/1.0	135.81 ± 7.09 ^c^	147.85 ± 3.28 ^c^
Isopimpinellin	0.5/1.0	54.97 ± 4.81 ^a^	29.09 ± 4.23 ^c^
	0.1/0.1	55.66 ± 11.44 ^a^	36.37 ± 2.66 ^c^
	1.0/1.0	75.58 ± 7.58 ^b^	84.48 ± 13.22 ^b^
Psoralen	0.5/1.0	222.29 ± 32.73 ^a^	208.31 ± 52.47 ^a^
	0.1/0.1	217.83 ± 12.56 ^a^	218.54 ± 9.97 ^a^
	1.0/1.0	125.88 ± 12.14 ^b^	119.58 ± 5.14 ^b^
Isoimperatorin	0.5/1.0	54.46 ± 7.97 ^ab^	42.09 ± 6.32 ^bc^
	0.1/0.1	50.28 ± 6.69 ^abc^	49.61 ± 7.78 ^abc^
	1.0/1.0	43.59 ± 2.41 ^abc^	50.56 ± 7.44 ^abc^
Total furanocoumarins	0.5/1.0	1169.05 ± 127.51 ^a^	840.89 ± 90.67 ^bcd^
	0.1/0.1	1124.62 ± 49.85 ^a^	921.05 ± 57.29 ^cd^
	1.0/1.0	756.29 ± 33.94 ^bc^	847.34 ± 46.07 ^bcd^

**Table 3:** Contents of furoquinoline alkaloids [mg/100 g DW] obtained from *R. chalepensis* cultures maintained in RITA^®^ bioreactors, depending on the duration of the growth cycle of culture (4 and 5 weeks) and LS medium variant NAA/BAP (0.5/1.0, 0.1/0.1, and 1.0/1.0 mg/L). Means of three replications ± SD. Letters denote homogeneous groups. Different letters indicate significant differences (*p* < 0.05).

Metabolite	LS Medium Variant	Content [mg/100 g DW]	
	NAA/BAP mg/L	4-Week Growth Cycle	5-Week Growth Cycle
Skimmianine	0.5/1.0	143.18 ± 4.89 ^a^	192.64 ± 19.26 ^d^
	0.1/0.1	122.44 ± 16.27 ^b^	291.59 ± 3.95 ^e^
	1.0/1.0	74.30 ± 4.99 ^c^	213.53 ± 3.41 ^f^
γ-fagarine	0.5/1.0	135.44 ± 9.47 ^ac^	98.97 ± 11.48 ^cd^
	0.1/0.1	186.88 ± 34.50 ^b^	143.84 ± 10.90 ^ac^
	1.0/1.0	124.47 ± 14.09 ^acd^	136.73 ± 15.36 ^ac^
7-isopentenyloxy-γ-fagarine	0.5/1.0	13.25 ± 2.94 ^ac^	13.04 ± 0.37 ^ac^
	0.1/0.1	16.79 ± 1.11 ^bc^	13.23 ± 2.47 ^ac^
	1.0/1.0	15.45 ± 0.49 ^abc^	15.45 ± 1.00 ^abc^
Total furoquinoline alkaloids	0.5/1.0	291.88 ± 9.01 ^ab^	304.64 ± 28.91 ^ab^
	0.1/0.1	326.10 ± 42.07 ^abe^	448.66 ± 12.86 ^d^
	1.0/1.0	214.22 ± 13.52 ^c^	365.70 ± 17.41 ^be^

## Data Availability

The data presented in this study are available on request from the corresponding author.

## Supplementary Materials

The following supporting information can be downloaded at https://www.mdpi.com/article/10.3390/life13020457/s1, Figure S1: Sample chromatogram of the extract from *Ruta chalepensis* in vitro cultures (LS NAA/BAP 0.1/0.1 mg/L medium, 5-week growth cycle): 1. psoralen, 2. xanthotoxin, 3. isopimpinellin, 4. skimmianine, 5. bergapten, 6. γ-fagarine, 7 isoimperatorin, 8. 7-isopentenyloxy-γ-fagarine.
