# Supplementary material for: Ruta chalepensis L. In Vitro Cultures as a Source of Bioactive Furanocoumarins and Furoquinoline Alkaloids
Source: Life (Basel). 2023 Feb 6;13(2):457. doi: 10.3390/life13020457 (PMC9959614; doi:10.3390/life13020457)
Supplement: Supplementary file 1 [file life-13-00457-s001.zip › life-2164687-supplementary.pdf]

SUPPLEMENTARY FILES

***Ruta chalepensis* L. *in vitro* cultures as a source of bioactive furanocoumarins and furoquinoline alkaloids**

I. HPLC ANALYSIS

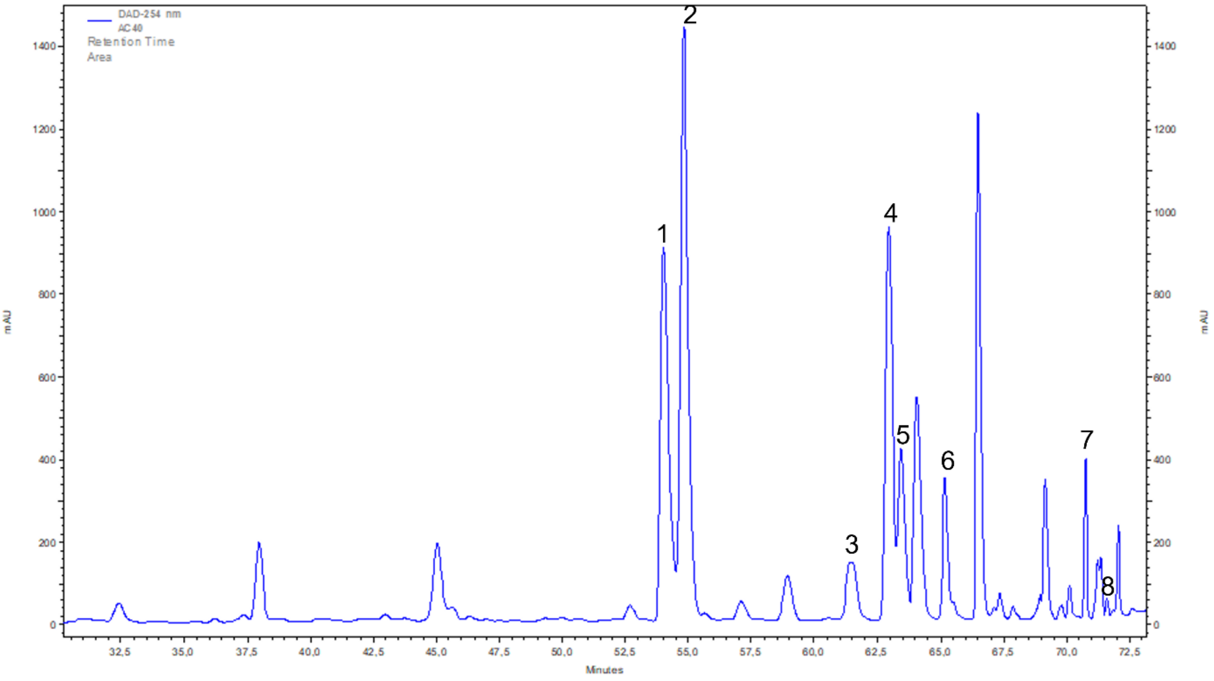

Figure S1 Sample chromatogram of the extract from *Ruta chalepensis in vitro* cultures (LS NAA/BAP 0.1/0.1 mg/L medium, 5-week growth cycle) 1. psoralen, 2. xanthotoxin, 3. isopimpinellin, 4. skimmianine, 5. bergapten, 6. γ-fagarine, 7. isoimperatorin, 8. 7-isopentenyloxy-γ-fagarine

2. STATISTICAL ANALYSIS

Statistical analysis of the content of individual secondary metabolites and comparison of homogeneous groups. Two-way analysis of variance (ANOVA), NIR poshoc test, dependent variable - metabolite content [mg/100 g DW], independent variables - culture cycle (4, 5 weeks) and LS medium variant (NAA/BAP 0.5/1.0, 0.1/0.1, 1.0/1.0 mg/L).

Xanthotoxin

Nr podkl.	Test NIR; zmienna mg/100 g DM (rch Rity publ Life do statystyki) Prawdopodobieństwa dla testów post-hoc Błąd: MS międzygrupowe = 1638,3, df = 12,000							
	week	LS medium variant	{1} 592,57	{2} 603,96	{3} 375,43	{4} 425,23	{5} 486,72	{6} 444,87
1	4	0,5/1		0,736191	0,000027	0,000278	0,007591	0,000767
2	4	0,1/0,1	0,736191		0,000016	0,000158	0,004013	0,000423
3	4	1,0/1,0	0,000027	0,000016		0,157745	0,005598	0,057434
4	5	0,5/1	0,000278	0,000158	0,157745		0,087480	0,563313
5	5	0,1/0,1	0,007591	0,004013	0,005598	0,087480		0,229473
6	5	1,0/1,0	0,000767	0,000423	0,057434	0,563313	0,229473	

Nr podkl.	Test NIR; zmienna mg/100 g DM (rch Rity publ Life do statystyki) Grupy jednorodne, alfa = ,05000 Błąd: MS międzygrupowe = 1638,3, df = 12,000					
	week	LS medium variant	mg/100 g DM Średnie	1	2	3
3	4	1,0/1,0	375,4320	****		
4	5	0,5/1	425,2270	****	****	
6	5	1,0/1,0	444,8696	****	****	
5	5	0,1/0,1	486,7153		****	
1	4	0,5/1	592,5681			****
2	4	0,1/0,1	603,9641			****

Bergapten

Nr podkl.	Test NIR; zmienna mg/100 g DM (rch Rity publ Life do statystyki) Prawdopodobieństwa dla testów post-hoc Błąd: MS międzygrupowe = 162,02, df = 12,000							
	week	LS medium variant	{1} 244,76	{2} 196,89	{3} 135,81	{4} 136,17	{5} 129,81	{6} 147,85
1	4	0,5/1		0,000605	0,000000	0,000000	0,000000	0,000001
2	4	0,1/0,1	0,000605		0,000075	0,000079	0,000031	0,000498
3	4	1,0/1,0	0,000000	0,000075		0,973043	0,574291	0,269432
4	5	0,5/1	0,000000	0,000079	0,973043		0,551969	0,283241
5	5	0,1/0,1	0,000000	0,000031	0,574291	0,551969		0,108256
6	5	1,0/1,0	0,000001	0,000498	0,269432	0,283241	0,108256	

Nr podkl.	Test NIR; zmienna mg/100 g DM (rch Rity publ Life do statystyki) Grupy jednorodne, alfa = ,05000 Błąd: MS międzygrupowe = 162,02, df = 12,000					
	week	LS medium variant	mg/100 g DM Średnie	1	2	3
5	5	0,1/0,1	129,8126	****		
3	4	1,0/1,0	135,8144	****		
4	5	0,5/1	136,1730	****		
6	5	1,0/1,0	147,8486	****		
2	4	0,1/0,1	196,8916		****	
1	4	0,5/1	244,7564			****

Isopimpinellin

Nr podkl.	week	LS medium variant	{1} 54,973	{2} 55,655	{3} 75,578	{4} 29,086	{5} 36,366	{6} 84,482
	Test NIR; zmienna mg/100 g DM (rch Rity publ Life do statystyki) Prawdopodobieństwa dla testów post-hoc Błąd: MS międzygrupowe = 69,025, df = 12,000							
1	4	0,5/1		0,921559	0,010323	0,002456	0,017834	0,000945
2	4	0,1/0,1	0,921559		0,012444	0,002047	0,014800	0,001129
3	4	1,0/1,0	0,010323	0,012444		0,000018	0,000087	0,213894
4	5	0,5/1	0,002456	0,002047	0,000018		0,304268	0,000003
5	5	0,1/0,1	0,017834	0,014800	0,000087	0,304268		0,000013
6	5	1,0/1,0	0,000945	0,001129	0,213894	0,000003	0,000013	

Nr podkl.	week	LS medium variant	mg/100 g DM Średnie	1	2	3
	Test NIR; zmienna mg/100 g DM (rch Rity publ Life do statystyki) Grupy jednorodne, alfa = ,05000 Błąd: MS międzygrupowe = 69,025, df = 12,000					
4	5	0,5/1	29,08603	****		
5	5	0,1/0,1	36,36648	****		
1	4	0,5/1	54,97331		****	
2	4	0,1/0,1	55,65548		****	
3	4	1,0/1,0	75,57795			****
6	5	1,0/1,0	84,48151			****

Psoralen

Nr podkl.	week	LS medium variant	{1} 222,29	{2} 217,83	{3} 125,88	{4} 208,31	{5} 218,54	{6} 119,58
	Test NIR; zmienna mg/100 g DM (rch Rity publ Life do statystyki) Prawdopodobieństwa dla testów post-hoc Błąd: MS międzygrupowe = 709,29, df = 12,000							
1	4	0,5/1		0,840918	0,000816	0,532554	0,866071	0,000494
2	4	0,1/0,1	0,840918		0,001172	0,669515	0,974361	0,000704
3	4	1,0/1,0	0,000816	0,001172		0,002573	0,001105	0,776756
4	5	0,5/1	0,532554	0,669515	0,002573		0,646558	0,001524
5	5	0,1/0,1	0,866071	0,974361	0,001105	0,646558		0,000665
6	5	1,0/1,0	0,000494	0,000704	0,776756	0,001524	0,000665	

Nr podkl.	week	LS medium variant	mg/100 g DM Średnie	1	2
	Test NIR; zmienna mg/100 g DM (rch Rity publ Life do statystyki) Grupy jednorodne, alfa = ,05000 Błąd: MS międzygrupowe = 709,29, df = 12,000				
6	5	1,0/1,0	119,5755		****
3	4	1,0/1,0	125,8819		****
4	5	0,5/1	208,3138	****	
2	4	0,1/0,1	217,8275	****	
5	5	0,1/0,1	218,5411	****	
1	4	0,5/1	222,2878	****	

Isoimperatorin

Nr podkl.	Test NIR; zmienna mg/100 g DM (rch Rity publ Life do statystyki)<br>Prawdopodobieństwa dla testów post-hoc<br>Błąd: MS międzygrupowe = 44,954, df = 12,000							
	week	LS medium variant	{1}<br>54,460	{2}<br>50,284	{3}<br>43,587	{4}<br>42,092	{5}<br>49,614	{6}<br>50,564
1	4	0,5/1		0,460295	0,070350	0,043264	0,393404	0,490253
2	4	0,1/0,1	0,460295		0,244713	0,160359	0,904580	0,960063
3	4	1,0/1,0	0,070350	0,244713		0,789320	0,292569	0,226654
4	5	0,5/1	0,043264	0,160359	0,789320		0,194548	0,147672
5	5	0,1/0,1	0,393404	0,904580	0,292569	0,194548		0,865100
6	5	1,0/1,0	0,490253	0,960063	0,226654	0,147672	0,865100	

Nr podkl.	Test NIR; zmienna mg/100 g DM (rch Rity publ Life do statystyki) Grupy jednorodne, alfa = ,05000 Błąd: MS międzygrupowe = 44,954, df = 12,000				
	week	LS medium variant	mg/100 g DM<br>Średnie	1	2
4	5	0,5/1	42,09158	****	
3	4	1,0/1,0	43,58737	****	****
5	5	0,1/0,1	49,61368	****	****
2	4	0,1/0,1	50,28395	****	****
6	5	1,0/1,0	50,56385	****	****
1	4	0,5/1	54,46002		****

Total furanocoumarins

Nr podkl.	Test NIR; zmienna mg/100 g DM (rch Rity publ Life do statystyki)<br>Prawdopodobieństwa dla testów post-hoc<br>Błąd: MS międzygrupowe = 5586,8, df = 12,000							
	week	LS medium variant	{1}<br>1169,0	{2}<br>1124,6	{3}<br>756,29	{4}<br>840,89	{5}<br>921,05	{6}<br>847,34
1	4	0,5/1		0,480636	0,000020	0,000166	0,001572	0,000197
2	4	0,1/0,1	0,480636		0,000059	0,000561	0,005935	0,000674
3	4	1,0/1,0	0,000020	0,000059		0,190904	0,019322	0,161555
4	5	0,5/1	0,000166	0,000561	0,190904		0,213594	0,917607
5	5	0,1/0,1	0,001572	0,005935	0,019322	0,213594		0,250392
6	5	1,0/1,0	0,000197	0,000674	0,161555	0,917607	0,250392	

Nr podkl.	Test NIR; zmienna mg/100 g DM (rch Rity publ Life do statystyki) Grupy jednorodne, alfa = ,05000 Błąd: MS międzygrupowe = 5586,8, df = 12,000					
	week	LS medium variant	mg/100 g DM<br>Średnie	1	2	3
3	4	1,0/1,0	756,294	****		
4	5	0,5/1	840,891	****	****	
6	5	1,0/1,0	847,339	****	****	
5	5	0,1/0,1	921,049		****	
2	4	0,1/0,1	1124,623			****
1	4	0,5/1	1169,046			****

Skimmianine

Nr podkl.	Test NIR; zmienna mg/100 g DM (rch Rity publ Life do statystyki)<br>Prawdopodobieństwa dla testów post-hoc<br>Błąd: MS międzygrupowe = 118,61, df = 12,000							
	week	LS medium variant	{1}<br>143,18	{2}<br>122,44	{3}<br>74,305	{4}<br>192,64	{5}<br>291,59	{6}<br>213,53
1	4	0,5/1		0,037861	0,000005	0,000124	0,000000	0,000004
2	4	0,1/0,1	0,037861		0,000157	0,000004	0,000000	0,000000
3	4	1,0/1,0	0,000005	0,000157		0,000000	0,000000	0,000000
4	5	0,5/1	0,000124	0,000004	0,000000		0,000000	0,036768
5	5	0,1/0,1	0,000000	0,000000	0,000000	0,000000		0,000001
6	5	1,0/1,0	0,000004	0,000000	0,000000	0,036768	0,000001	

Nr podkl.	Test NIR; zmienna mg/100 g DM (rch Rity publ Life do statystyki) Grupy jednorodne, alfa = ,05000 Błąd: MS międzygrupowe = 118,61, df = 12,000								
	week	LS medium variant	mg/100 g DM Średnie	1	2	3	4	5	6
3	4	1,0/1,0	74,3046	****					
2	4	0,1/0,1	122,4374		****				
1	4	0,5/1	143,1838			****			
4	5	0,5/1	192,6365				****		
6	5	1,0/1,0	213,5264					****	
5	5	0,1/0,1	291,5940						****

γ-fagarine

Nr podkl.	Test NIR; zmienna mg/100 g DM (rch Rity publ Life do statystyki)<br>Prawdopodobieństwa dla testów post-hoc<br>Błąd: MS międzygrupowe = 327,48, df = 12,000							
	week	LS medium variant	{1}<br>135,44	{2}<br>186,88	{3}<br>124,47	{4}<br>98,968	{5}<br>143,84	{6}<br>136,73
1	4	0,5/1		0,004537	0,472114	0,029574	0,580184	0,932002
2	4	0,1/0,1	0,004537		0,001182	0,000067	0,013018	0,005329
3	4	1,0/1,0	0,472114	0,001182		0,109960	0,214419	0,422985
4	5	0,5/1	0,029574	0,000067	0,109960		0,010331	0,025202
5	5	0,1/0,1	0,580184	0,013018	0,214419	0,010331		0,638921
6	5	1,0/1,0	0,932002	0,005329	0,422985	0,025202	0,638921	

Nr podkl.	Test NIR; zmienna mg/100 g DM (rch Rity publ Life do statystyki) Grupy jednorodne, alfa = ,05000 Błąd: MS międzygrupowe = 327,48, df = 12,000					
	week	LS medium variant	mg/100 g DM Średnie	1	2	3
4	5	0,5/1	98,9683		****	
3	4	1,0/1,0	124,4725	****	****	
1	4	0,5/1	135,4423	****		
6	5	1,0/1,0	136,7298	****		
5	5	0,1/0,1	143,8421	****		
2	4	0,1/0,1	186,8777			****

7-isopentenyloxy-γ-fagarine

Nr podkl.	week	LS medium variant	{1} 13,249	{2} 16,786	{3} 15,448	{4} 13,039	{5} 13,227	{6} 15,449
	Test NIR; zmienna mg/100 g DM (rch Rity publ Life do statystyki) Prawdopodobieństwa dla testów post-hoc Błąd: MS międzygrupowe = 2,8911, df = 12,000							
1	4	0,5/1		0,025583	0,139223	0,882300	0,987788	0,139060
2	4	0,1/0,1	0,025583		0,354215	0,019354	0,024858	0,354559
3	4	1,0/1,0	0,139223	0,354215		0,108317	0,135706	0,999441
4	5	0,5/1	0,882300	0,019354	0,108317		0,894377	0,108186
5	5	0,1/0,1	0,987788	0,024858	0,135706	0,894377		0,135547
6	5	1,0/1,0	0,139060	0,354559	0,999441	0,108186	0,135547	

Nr podkl.	week	LS medium variant	mg/100 g DM Średnie	1	2
	Test NIR; zmienna mg/100 g DM (rch Rity publ Life do statystyki) Grupy jednorodne, alfa = ,05000 Błąd: MS międzygrupowe = 2,8911, df = 12,000				
4	5	0,5/1	13,03901	****	
5	5	0,1/0,1	13,22728	****	
1	4	0,5/1	13,24898	****	
3	4	1,0/1,0	15,44778	****	****
6	5	1,0/1,0	15,44877	****	****
2	4	0,1/0,1	16,78568		****

Total furoquinoline alkaloids

Nr podkl.	week	LS medium variant	{1} 291,88	{2} 326,10	{3} 214,22	{4} 304,64	{5} 448,66	{6} 365,70
	Test NIR; zmienna mg/100 g DM (rch Rity publ Life do statystyki) Prawdopodobieństwa dla testów post-hoc Błąd: MS międzygrupowe = 556,35, df = 12,000							
1	4	0,5/1		0,100877	0,001663	0,519859	0,000003	0,002380
2	4	0,1/0,1	0,100877		0,000084	0,287040	0,000036	0,062164
3	4	1,0/1,0	0,001663	0,000084		0,000519	0,000000	0,000004
4	5	0,5/1	0,519859	0,287040	0,000519		0,000007	0,008061
5	5	0,1/0,1	0,000003	0,000036	0,000000	0,000007		0,001018
6	5	1,0/1,0	0,002380	0,062164	0,000004	0,008061	0,001018	

Nr podkl.	week	LS medium variant	mg/100 g DM Średnie	1	2	3	4
	Test NIR; zmienna mg/100 g DM (rch Rity publ Life do statystyki) Grupy jednorodne, alfa = ,05000 Błąd: MS międzygrupowe = 556,35, df = 12,000						
3	4	1,0/1,0	214,2248			****	
1	4	0,5/1	291,8751	****			
4	5	0,5/1	304,6438	****			
2	4	0,1/0,1	326,1008	****	****		
6	5	1,0/1,0	365,7049		****		
5	5	0,1/0,1	448,6634				****
